# Aortic remodelling and late outcomes following thoracic endovascular repair with a bare-metal stent distal extension among patients with complicated type-B aortic dissection

**DOI:** 10.1093/icvts/ivac244

**Published:** 2022-09-23

**Authors:** Isaac Wamala, Mir Timo Zadegh Nazari-Shafti, Roland Heck, Adam Penkalla, Matteo Montagner, Steven J Staffa, Volkmar Falk, Semih Buz

**Affiliations:** Department of Cardiothoracic and Vascular Surgery, German Heart Center Berlin, Berlin, Germany; Department of Cardiovascular Surgery, Charité Universitätsmedizin Berlin, Berlin, Germany; Department of Cardiothoracic and Vascular Surgery, German Heart Center Berlin, Berlin, Germany; Department of Cardiothoracic and Vascular Surgery, German Heart Center Berlin, Berlin, Germany; Department of Cardiothoracic and Vascular Surgery, German Heart Center Berlin, Berlin, Germany; Department of Cardiothoracic and Vascular Surgery, German Heart Center Berlin, Berlin, Germany; Department of Anesthesiology, Critical Care and Pain Medicine, Boston Children’s Hospital, Boston, MA, USA; Department of Cardiothoracic and Vascular Surgery, German Heart Center Berlin, Berlin, Germany; Department of Cardiovascular Surgery, Charité Universitätsmedizin Berlin, Berlin, Germany; Deutsches Zentrum für Herz-Kreislauf Forschung (DHZK), Berlin, Germany; Department of Health Sciences and Technology, ETH Zürich, Zurich, Switzerland; Department of Cardiothoracic and Vascular Surgery, German Heart Center Berlin, Berlin, Germany

**Keywords:** Type B aortic dissection, TEVAR, PETTICOAT technique

## Abstract

**OBJECTIVES:**

The goal of this study was to describe the factors affecting mid and late aortic remodelling following thoracic endovascular aortic repair with the PETTICOAT (Provisional Extension To Induce Complete Attachment) technique among patients with complicated acute or subacute type B aortic dissection.

**METHODS:**

A retrospective single-centre study that evaluates clinical and morphological outcomes among 65 consecutive patients. The area and diameter of the true and false lumen, overall aortic diameter and false lumen perfusion were evaluated.

**RESULTS:**

Concomitant direct visceral artery stenting was successfully conducted in 32 (49%) patients. There was one (1.5%) postoperative stroke; three (4.6%) patients developed spinal cord ischaemia; two (3%) patients suffered retrograde type A dissection; and two (3%) patients had mesenteric ischaemia, despite successful reperfusion, that required a bowel resection. Median postoperative follow-up was 63.1 (interquartile range, 32.1– 91.8) months. The probability of survival was 96.9% [95% confidence interval (CI) 88.3%–99.2%] at 30 days, 93.9% (95% CI 84.4%–97.6%) at 1 year, 78.0 (95% CI 64.2%–87.0%) at 5 years and 72.8% (95% CI at 57.9%–83.2%) at 10 years postoperatively. There was a statistically significant postoperative increase in true-lumen area, diameter and true-lumen index in all five aortic levels measured. Complete false lumen (FL) thrombosis at the coeliac trunk, renal arteries and aortic bifurcation levels was observed in 47%, 15% and 24% of patients at midterm (6–15 months) and in 29%, 21% and 29% on late (later than 21 months) computed tomography angiograms (CTA). Persistent false lumen (FL) perfusion at the coeliac level on midterm CTA was associated with a larger extent of late aortic growth (*P* = 0.042) and was, in the majority of cases, caused by iliac re-entries either alone (28.57) or in combination with visceral and lumbar (28.57%) or distal aortic (10.71%) re-entries. A larger abdominal aortic diameter at midterm was associated with an increased probability of distal aortic reinterventions (hazard ratio 7.26, 95% CI 2.41–21.9, *P* < 0.001).

**CONCLUSIONS:**

Persistent FL perfusion of the distal aorta at midterm following TEVAR with the PETTICOAT technique among patients with acute and subacute type B dissection is caused mainly by iliac, visceral, lumber and distal aorta re-entries. Patients with persistent FL perfusion have an increased risk of aortic aneurysmal growth at late follow-up.

**Figure:**
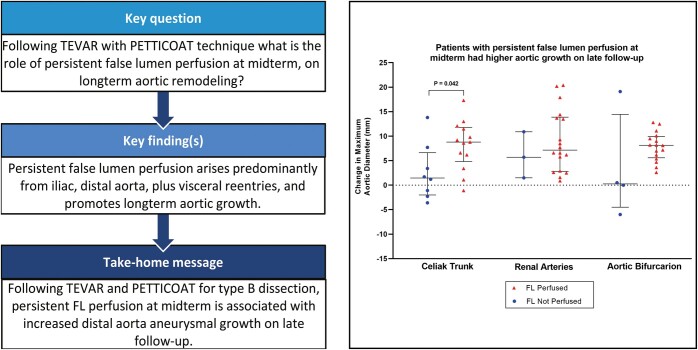

## INTRODUCTION

Thoracic endovascular repair (TEVAR) combined with medical optimization is recommended among patients with complicated type-B aortic dissection [1]. By closing the proximal entry tear, TEVAR improves true lumen (TL) perfusion and obliterates antegrade flow in the false lumen (FL). These changes result in favourable aortic remodelling in the mid to long term and prevent disease progression [2, 3]. However, aneurysmal degeneration and new stent-induced entry tears, especially of the aorta distal to the thoracic stent graft, remain issues of concern on long-term follow-up [4–6].

The PETTICOAT technique of scaffolding the aortic intimal lamella distal to the TEVAR stent graft using a bare-metal stent (Provisional Extension To Induce Complete Attachment) has been described [7, 8]. Modifications to this technique including preplacement of the distal scaffolding stent [9] or extended endovascular repair of the aorta (extended PETTICOAT) [10, 11] are feasible and safe.

The current literature indicates that in selected patients, the PETTICOAT technique compared to TEVAR alone provides a beneficial effect for positive aortic remodelling [12–17]. The key factor appears to be patient selection [18]. Clearly, most surgeons do not conduct an additional PETTICOAT procedure when TEVAR alone will do. Conversely, many surgeons use the PETTICOAT procedure in cases with either persistent TL collapse, radiological signs of dynamic malperfusion or lack of brisk flow into the visceral arteries, after correct placement of the TEVAR stent graft. Whether to perform this procedure at the same time or to wait for clinical signs of malperfusion is controversial.

The possible reasons why some patients undergoing the PETTICOAT technique achieve positive remodelling while others have persistent TL perfusion and late aortic growth remain largely unexamined. We evaluated factors affecting mid- and late-term aortic remodelling.

## PATIENTS AND METHODS

The ethics committee of the Charité–Universitätsmedizin Berlin approved the retrospective study (ethics approval number EA2/104/20).

The patient cohort included all consecutive patients with complicated acute (within 2 weeks of dissection occurrence) and subacute (greater than 2 weeks but less than 6 weeks after dissection occurrence) Stanford type B aortic dissection who underwent TEVAR with the PETTICOAT technique between September 2009 and March 2019 at the German Heart Center Berlin.

The study is a single-centre retrospective review of the clinical and morphologic outcomes following TEVAR with the PETTICOAT technique among these patients. Complicated dissections were those with either aortic rupture, rapidly expanding false lumen, acute increase in back or chest pain (probably indicating tear propagation or expanding false lumen) and those with distal malperfusion.

All patients underwent TEVAR with a distal bare-metal extension using the E-XL (Jotec GmbH, Germany) stent [7]. The length and diameter of the thoracic stent graft used for TEVAR was chosen according to the morphology of the affected aorta with minimal oversizing and ideally at least 25-mm proximal and distal landing zones. The decision to perform the PETTICOAT procedure was based on intraoperative angiographic evaluation. If, despite closure of the proximal entry tear, the TL in the abdominal aorta remained collapsed or if there was considerable retrograde FL flow (floating intimal lamella), dynamic malperfusion or otherwise lack of prompt filling of the visceral or iliac branches, the PETTICOAT technique was used. We typically create an overlap of 2 to 3 cm between the covered stent graft and the bare-metal stent or between the bare-metal stent components if more than one is used. The diameter of the bare-metal stent was based on the distal diameter of the already implanted thoracic stent graft as well as on the maximum aortic diameter of the distal landing zone for the bare-metal stent. Based on the foregoing considerations, we have used both straight and tapered bare-metal stents. In the beginning of our experience, some bare-metal distal extensions were implanted in the thoracoabdominal region extending distally to just above the coeliac trunk. More recently, we favour extending distally to the infrarenal aorta.

The survival status of the patients was ascertained by querying the state civil register. Clinical follow-up was conducted by reviewing the postoperative outpatient visits or by contacting the patients directly via telephone and was updated up to the end of 2020.

### Morphological analysis and computed tomography angiography measurements

**Figure 1:**
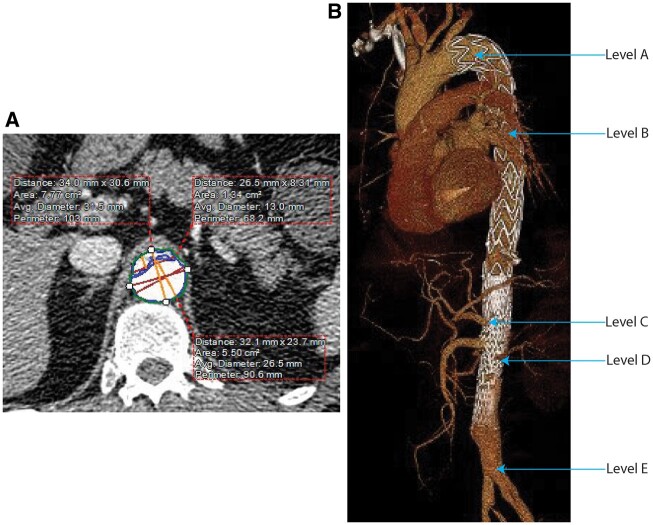
**(A)** The measurement of true lumen, false lumen and maximal aortic diameter dimensions. **(B)** The aortic levels where measurements were made.

Contrast computed tomography angiograms (CTA) of the aorta taken at 4 time points—immediately preoperatively, early postoperatively (within 2 weeks), between 6 and 15 months (midterm CTA) and at greater than 21 months (late CTA)—were analysed. Due to the retrospective nature of the study, these intervals were chosen to take into account the guideline-recommended follow-up schedules and the real-world scenarios in which follow-up appointments happened not exactly on the 1- or 2-year mark but rather according to patient availability. Follow-up CTA were only analysed prior to any reintervention on the aorta. CTA measurements were made at 5 levels of the aorta: level A—immediately distal to the left subclavian artery; level B—at the level of the tracheal carina; level C—immediately proximal to the coeliac trunk; D—immediately distal to the most distal renal artery; and level E—at the aortic bifurcation (Fig. 1). At each level, the following measurements were made: true lumen area (TLA); false lumen area (FLA); true lumen index (TLI =TLA/TLA+FLA); true lumen average diameter (TLD); false lumen average diameter and maximal aortic diameter (MAD). All of the above measurements were performed with orientation to the aortic centreline. TLA, FLA, TLI, TLD and false lumen average diameter measurements were made only of the aortic lumen and excluded the aortic wall. The MAD measurement included the aortic wall. All CTA were of the arterial phase, and measurements were made using automated gradient segmentation. All measurements were made by a single observer (IW) using Aquarius Nutrition software (TeraRecon GmbH, Frankfurt am Main, Germany). The measurement protocol was predefined with the required parameters and thresholds. The observer was responsible for choosing the exact axial slide to be measured, demarcating the true and false lumens, and checking that the automatic centreline detection and measurements were executed without errors. As detailed previously, the axial measurement levels were predefined based on anatomical parts of the image, thereby further reducing chances of operator bias.

### Quantitative grading of aortic remodelling

Two surgeons (IW and SB) reviewed all the CTA first separately and then in several mutual sittings, to reconcile their qualitative grading of the aortic remodelling, FL perfusion and the overall implant status at the different time points. The reported grades represent a consensus grade. Complete FL collapse or thrombosis was graded as being the complete disappearance of the FL or the complete thrombosis without any evidence of FL contrast in the concerned aortic section. Additionally, a qualitative grading of aortic remodelling on postoperative CTs as either complete positive remodelling or not, for the thoracic (above the coeliac artery), thoracoabdominal (coeliac artery to the renal arteries) and the infrarenal aorta, was conducted. For cases with persistent FL perfusion in the midterm or long term, a determination was made regarding the main cause of the perfusion.

### Statistical analyses

Continuous variables are summarized as median and interquartile range whereas categorical variables are characterized as frequencies and percentages. The Wilcoxon signed rank test was used to compare CTA parameters for aortic remodelling at the different time points, with a Bonferroni-adjusted *P* < 0.0083 implemented to determine statistical significance accounting for multiple comparisons and to preserve family-wise type 1 error of 0.05. For each CTA parameter, all pairwise time-point comparisons were made for preoperative, postoperative, 9–15 month and >21 month time points. Correlation between CTA parameters was estimated using Spearman correlation coefficients. Kaplan–Meier curves were constructed to estimate overall survival, reintervention and event-free survival (freedom from death, stroke, abdominal or limb ischaemia, paraplegia and new and persisting kidney injury) with 95% confidence bands calculated using Greenwood’s formula. Univariable Cox regression analysis was used to evaluate the association between preoperative and procedural risk variables to overall survival. Competing risks regression analysis using the Fine and Gray model was implemented to analyse the adjusted association between CTA measurements at the midterm time point and later reintervention while accounting for death as a competing event. Results from time-to-event regression analyses are reported as adjusted hazard ratios with 95% confidence intervals and *P* values. Receiver operating characteristic (ROC) curve analysis was implemented to determine the optimal cut-point for a given parameter in predicting reintervention using Youden’s J index to maximize the sum of sensitivity and specificity. ROC analysis results are summarized by the area under the curve (AUC). A two-tailed *P* < 0.05 was used to determine statistical significance.

## RESULTS

### Patients

Of 146 patients with acute or subacute type B aortic dissection undergoing TEVAR during this time, the PETTICOAT technique was used in 65 patients. The patient and procedure details are summarized in Table 1. The mean age was 56 (± 11) years. A total of 54 (83.1%) patients had an acute dissection whereas 11 (16.9%) had a subacute dissection. The indications for TEVAR were visceral malperfusion (29.2%), iliac malperfusion (7.7%), rapid increase in FL size or refractory pain (30.8%) and aortic rupture (4.6%). Eighteen (27.7%) patients had more than one indication including three (4.6%) patients with preoperative paraplegia. Only three (4.6%) patients had exclusive TL perfusion to all aortic branch arteries. One patient presenting with acute coeliac artery obstruction had a coeliac artery stent placed 2 weeks prior to TEVAR; another patient had a femoral cross-over bypass to treat limb ischaemia prior to being referred to our clinic.

**Table 1:** Patient and procedure characteristics

Parameter	Patients (N = 65)
Age	56 (± 11)
Female gender	17 (26.2%)
Hypertension	55 (84.6%)
Traumatic dissection	2 (3.1%)
Marfan syndrome	5 (7.7%)
Dissection acuity	
Acute	54 (83.1%)
Subacute	11 (16.9%)
Indications for TEVAR	
Visceral malperfusion	19 (29.2%)
Iliac malperfusion	5 (7.7%)
Rapid increase in FL size or refractory pain	20 (30.8%)
Aortic rupture	3 (4.6%)
Multiple	18 (27.7%)
Aortic debranching	
Left carotid-subclavian bypass	22 (33.8%)
Left carotid artery transposition	2 (3.1%)
Left subclavian overstenting w/o debranching	5 (7.7%)
Proximal landing zone	
1	2 (3.1%)
2	27 (41.5%)
3	34 (52.3%)
4	2 (3.1%)
Proximal oversizing percentage (median)	6.4 % [2.6 , 10.1]
Additional stents in aortic branches	
Coeliac trunk	4 (6.2%)
Renal artery	18 (27.7%)
Iliac artery	5 (7.7%)
Multiple branch stents	5 (7.7%)
Stented aorta (mm)	342.5 [308,25 -387,75]
Length of covered stent graft (mm)	228 [200, 253]
Multiple covered segments used	9 (13.8%)
Multiple E-XL segments used	6 (9.2%)
Length between covered segment and coeliac trunk (mm)	24 [44, 77]
Distal extent of bare-metal stent	
Supracoeliac	6 (9.2%)
Suprarenal	8 (12.3)
Infrarenal	50 (76.9%)
Dose area product (μGray/m^2^)	47223 [27720.5, 73654]
Fluoroscopy time (min)	15.6 [10.6, 23,25]
Amount of contrast (ml)	180 [140, 250]

FL: false lumen; TEVAR: thoracic endovascular aortic repair; w/o: without.

**Table 2:** Early and late outcomes

Parameters	Patients (N = 65)
30-Day deaths	2 (3.08%)
1-Year deaths	4 (6.15%)
Event frequency (by latest follow-up examination)	
Stroke	1 (1.5%)
Retrograde type A	2(3%)
Localized type A dissection	1(1.5%)
Mesenteric infarct or persistent visceral ischaemia	2 (3%)
Spinal cord ischaemia	3 (4.6%)
Popliteal artery thrombosis	1 (1.5%)
Late stent damage or dislocation	4(6.2%)
Reinterventions (by latest follow-up examination)	
Ascending aorta replacement	3 (4.6%)
Proximal endovascular extension with CS bypass or LCC transposition	6 (9.3%)
Distal endovascular reintervention or EVAR	5 (7.69%)
Thoracoabdominal aortic replacement	5 (7.69%)

CS: carotid-subclavian; EVAR: endovascular aneurysm repair; LCC: left common carotid

**Table 3:** Changes in aortic morphologic measurements over time

CT Measurement	Preop (n = 65)	Post-op (n = 65)	1 year (n = 34)	Late follow-up (n = 25)	*P*1	*P*2	*P*3	*P*4	*P*5	*P*6
TLA (cm^2^)										
A	5.04 (2.83, 6.49)	6.53 (5.07, 7.5)	7.82 (5.87, 8.98)	7.54 (6.59, 8.92)	<0.001^*^	<0.001^*^	<0.001^*^	0.002^*^	<0.001^*^	0.062
B	3.25 (2.25, 4.74)	6.55 (5.27, 7.83)	8.26 (7.11, 9.97)	8.06 (7.04, 10.4)	<0.001^*^	<0.001^*^	<0.001^*^	<0.001^*^	<0.001^*^	0.092
C	1.79 (1.23, 3.02)	5.27 (4.48, 5.96)	5.4 (4.77, 6.41)	5.83 (4.93, 6.55)	<0.001^*^	<0.001^*^	<0.001^*^	0.027	<0.001^*^	0.024
D	1.51 (0.99, 2.44)	3.48 (2.63, 4.46)	3.76 (2.68, 5)	3.49 (2.57, 4.46)	<0.001^*^	<0.001^*^	<0.001^*^	0.003^*^	0.004^*^	0.179
E	1.31 (0.85, 2.82)	2.45 (1.86, 3.52)	2.42 (2.13, 3.67)	2.63 (2.1, 3.7)	<0.001^*^	<0.001^*^	<0.001^*^	0.108	0.07	0.032
TLD (mm)										
A	24.9 (19, 28.4)	28.6 (25.2, 30.9)	31.1 (27.1, 33.5)	31.9 (29, 35.6)	<0.001^*^	<0.001^*^	<0.001^*^	0.013	<0.001^*^	0.001^*^
B	20.9 (16.9, 24.7)	28.7 (25.8, 31.3)	32.5 (30.1, 35.6)	32 (29.9, 35.5)	<0.001^*^	<0.001^*^	<0.001^*^	<0.001^*^	<0.001^*^	0.219
C	15.1 (12.5, 19.6)	25.9 (23.9, 27.5)	26.2 (24.7, 28.6)	27 (24.4, 28.9)	<0.001^*^	<0.001^*^	<0.001^*^	0.042	0.002^*^	0.022
D	14.3 (11.2, 17.6)	21 (18.5, 23.8)	22.1 (18.7, 25.2)	20.8 (17.4, 23.6)	<0.001^*^	<0.001^*^	<0.001^*^	0.001^*^	0.035	0.492
E	13.1 (10.6 (19.3)	17.6 (15.3, 20.6)	17.6 (16.5, 21.6)	18.3 (16.4, 21.7)	<0.001^*^	<0.001^*^	0.002^*^	0.05	0.067	0.045
MAD (mm)										
A	37.2 (32.1, 43.5)	31.9 (28.9, 35.7)	33.7 (30.2, 37.4)	37.7 (33.6, 42.3)	<0.001^*^	<0.001^*^	<0.001^*^	0.545	<0.001^*^	<0.001^*^
B	38.7 (33, 43.2)	40.5 (36.6, 45.7)	37.7 (32.9, 43.5)	38.6 (33, 43.8)	<0.001^*^	0.826	0.363	0.049	0.353	0.147
C	33.2 (29.7, 36.2)	34.2 (30, 37.1)	34 (31.3, 38.2)	39.4 (31.9, 43.8)	0.081	0.126	<0.001^*^	0.201	0.002^*^	<0.001^*^
D	26.6 (24.2, 30.5)	27.5 (25.3, 30)	30.8 (28.4, 33.5)	34.4 (27.9, 38.2)	0.111	<0.001^*^	<0.001^*^	<0.001^*^	<0.001^*^	<0.001^*^
E	24.9 (22.1, 28.1)	25.6 (23.2, 28.8)	27.1 (25, 30.4)	30.7 (27, 37.6)	0.079	<0.001^*^	<0.001^*^	0.01^*^	<0.001^*^	<0.001^*^
FL complete thrombosis										
A		48/63 (76.2%)	31/32 (96.9%)	22/25 (88%)				0.999	0.999	0.999
B		41/65 (63.1%)	32/34 (94.1%)	23/25 (92%)				0.139	0.487	0.999
C		14/65 (21.5%)	16/34 (47.1%)	7/24 (29.2%)				0.999	0.076	0.325
D		11/65 (16.9%)	5/34 (14.7%)	5/24 (20.8%)				0.015	0.38	0.041
E		9/65 (13.8%)	8/33 (24.2%)	7/24 (29.2%)				0.241	0.017	0.032
TLI										
A	0.49 (0.31, 1)	1 (1, 1)	1 (1, 1)	1 (0.75, 1)	<0.001^*^	<0.001^*^	0.019	0.141	0.486	0.016
B	0.32 (0.26, 0.4)	0.55 (0.43, 0.69)	1 (1, 1)	1 (1, 1)	<0.001^*^	<0.001^*^	<0.001^*^	<0.001^*^	<0.001^*^	0.031
C	0.26 (0.19, 0.42)	0.67 (0.55, 0.75)	0.63 (0.52, 1)	0.53 (0.45, 1)	<0.001^*^	<0.001^*^	<0.001^*^	0.402	0.889	0.145
D	0.34 (0.2, 0.47)	0.65 (0.54, 0.76)	0.61 (0.51, 0.7)	0.57 (0.35, 0.71)	<0.001^*^	<0.001^*^	<0.001^*^	0.655	0.296	0.067
E	0.34 (0.21, 0.52)	0.58 (0.47, 0.68)	0.53(0.43, 0.69)	0.42 (0.35, 0.67)	<0.001^*^	<0.001^*^	0.111	0.192	0.006^*^	0.171

^*^ statistically significant (p<0.0083).

CT: computed tomography; FL: false lumen; MAD: maximal aortic diameter; Post-op: postoperative; Pre-op: preoperative; TLA: true lumen area; TLD: true lumen diameter; TLI: true lumen diameter.

### Details of the procedure

Relay NBS thoracic aortic stents (Bolton Medical, Terumo Aortic, Sunrise, FL, USA) were implanted in 43 (66.2%) patients, whereas 22 (33.8%) patients received EVITA thoracic aortic stents (JOTEC GmbH, Hechingen, Germany). Additional visceral and iliac artery stents were implanted among 32 (49%) patients (Table 1). The proximal landing area was in aortic zones 1 and 2 (3.1%) patients and was in zone 2 in 27 (41.5%) patients. The median proximal oversizing was 6.4%. The distal end of the E-XL stent (JOTEC) was supracoeliac in 6 (9.2%) patients, suprarenal in 8 (12.3%) patients and infrarenal in 50 (76.9%) patients.

### Clinical outcomes

Early and late outcomes are summurized in table 2. Survival follow-up was complete in 64 (98.4%) patients. In one patient who lived outside Europe, survival follow-up beyond the 1-year postoperative clinic visit was not possible. Updated clinical follow-up was complete in 59 (90.1%) patients whereas in 6 patients no updated information could be collected either due to changed contact information or to non-response to follow-up queries. Follow-up in these six patients ranged between 1 month and 28 months postoperatively. Overall, the median follow-up was 63.1 (interquartile range, 32.1–91.8) months.

### 30-Day outcomes

Technically successful TEVAR was achieved in all patients. Eight (12.3%) minimal primary leaks were noted on intraoperative angiography; these were accepted in view of the overall clinical situation. In all but one patient with failed revascularization of the left renal artery, complete visceral and iliac perfusion was successfully restored. One patient suffered a postoperative ischaemic pontine infarction with left-sided hemiplegia and aphasia but recovered and had no residual symptoms at follow-up. One patient with a pre-existing ascending aortic aneurysm suffered a local type-A aortic dissection (not continuous with the descending aorta) 6 days after TEVAR. Spinal cord ischaemia was observed in three (4.6%) patients. Two (3.1%) patients suffered limb ischaemia. Acute kidney injury (according to the RIFLE criteria) persisted up to discharge among four (6.2%) patients. No cases of new dialysis dependency occurred at discharge from the hospital. Bowel resection or stoma creation was required in two patients. Two patients (3.1%) died within 30 days postoperatively. Both patients had significant visceral malperfusion and acidosis prior to the TEVAR and died of multiorgan failure despite successful reestablishment of visceral perfusion.

### Late clinical events

Two (3.1%) patients suffered retrograde Stanford type-A aortic dissections 6 months and 23 months postoperatively, one of which followed a re-TEVAR and proximal extension. On follow-up, two stent graft kinks and one type-3 leak with stable extravasation between the covered stent graft and the E-XL stent were observed. None of these required reintervention. There were no cases of endograft infection on follow-up.

### Survival

**Figure 2:**
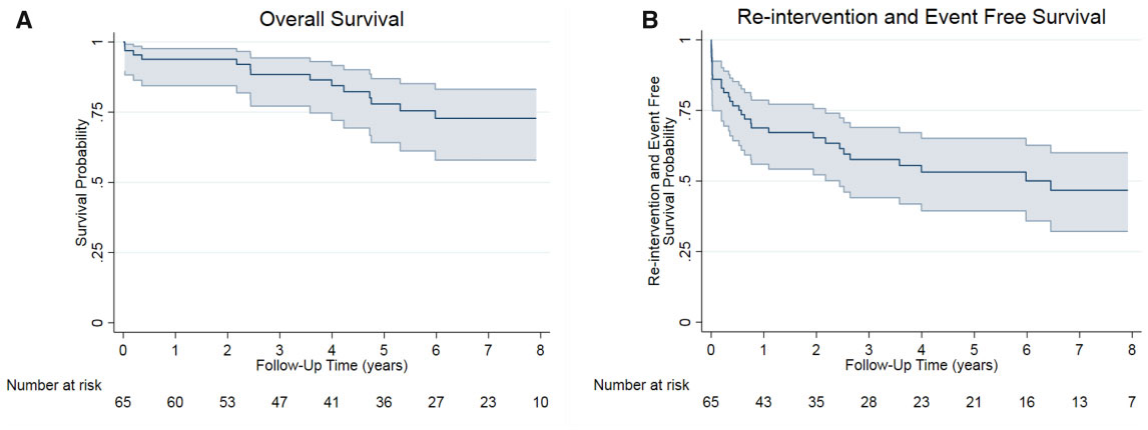
Long-term survival **(A)** and reintervention-free survival **(B)** following the Petticoat technique.

The overall probability of survival was 96.9% (95% CI 88.3%–99.2%) at 30 days, 93.9% (95% CI 84.4%–97.6%) at 1 year, 78.0 (95% CI 64.2%–87.0%) at 5 years and 72.8% (95% CI at 57.9%–83.2%) at 10 years postoperatively (Fig. 2A). The diameter of the proximal landing zone (HR 1.18, 95% CI 1.04–1.34, *P* = 0.011) and the proximal diameter [hazard ratio (HR) 1.18, CI 1.02–1.37, *P* = 0.025] of the stent graft required were the only parameters significantly associated with death on univariable Cox regression analysis.

### Reinterventions

Three (4.6%) patients underwent ascending aortic replacement due to type-A aortic dissections at 6 days, 6 months and 23 months postoperatively. Another patient underwent ascending aorta and hemiarch replacement at 66 months due to an aortic arch aneurysm, having previously undergone proximal and distal endovascular extensions at 7 months postoperatively. At the latest follow-up, 7 (10.8%) patients had undergone proximal endovascular repair extensions. Six of these, with type IA endoleaks, underwent aortic debranching (left carotid subclavian bypass in five patients and left carotid and subclavian transposition in one patient). The seventh patient had a type II endoleak via the subclavian artery stump following carotid-subclavian bypass and TEVAR, requiring initial placement of an Amplatzer plug (Abbott GmbH, Germany) in the subclavian stump and, subsequently, proximal endovascular extension. Four (6.2%) patients underwent thoracoabdominal aortic replacement due to aneurysm progression at 8, 31, 51 and 77 months, respectively; two (3.1%) patients underwent abdominal aortic replacement at 9 and 38 months of follow-up. Four (6.15%) patients underwent EVAR or distal endovascular interventions due to distal re-entries. Two of the patients underwent endovascular closure of iliac re-entries at 1 and 5 months postoperatively. One (1.5%) patient underwent distal endovascular extension at 7 months, and another patient underwent EVAR at 22 months. Overall reintervention- and event-free survival was 86.0% (95% CI 74.9%–92.5%) at 30 days, 68.8% (95% CI 56.0%–78.6%) at 1 year, 53.2% (95% CI 39.4%–65.2%) at 5 years and 46.7% (95% CI 32.2%–60.0%) at 10 years (Fig. 2B). On univariable competing risks regression analysis, the risk of aortic reintervention was associated with younger age (HR 0.95, 95% CI 0.91–0.99, *P* = 0.029) and higher volume of contrast requirement at the initial TEVAR (HR 1.01, 95% CI 1.01–1.02, *P* = 0.032). Patients with visceral malperfusion (HR 2.66, 95% CI 0.85–8.34, *P* = 0.093) and patients who required coeliac artery stents (HR 4.21, 95% CI 0.81–21.9, *P* = 0.087) or iliac artery stents (HR 4.81, 95% CI 0.86–26.9, *P* = 0.074) had a higher tendency towards aortic reintervention. Comparing suprarenal versus infrarenal distal extents of the E-XL stent, suprarenal extent is associated with 3 times (95% CI: 0.97, 9.26; *P* = 0.056) the risk of distal reintervention. Eight (12.3%) patients required repeated vascular reinterventions with time.

### Aortic remodelling

**Figure 3:**
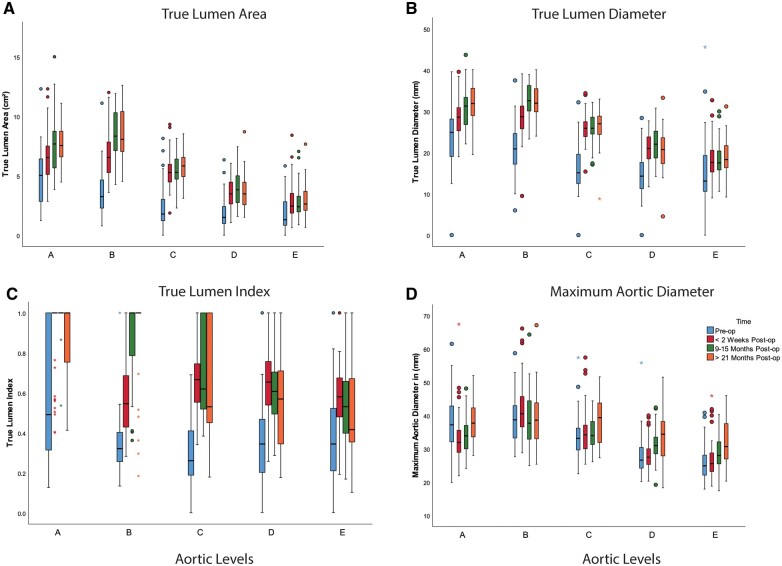
The changes in the true lumen area **(A)** true lumen diameter **(B)**, true lumen index **(C)** and maximal aortic diameter **(E)** over time at the different aortic levels. Pre-op: preoperatively; Post-op: postoperatively.

CTA were available for all patients at the preoperative and early postoperative time points, for 34 (52%) patients at midterm and 25 (38.5%) patients at the late follow-up. The changes in the aortic morphologic parameters over time are illustrated in Fig. 3A-C and summarized in Table 3. There was a statistically significant increase in TLA, TLD and TLI in all 5 aortic levels measured, which increase was maintained at midterm and at late follow-up. There was a significant growth in MAD over time. In the majority of cases, this growth did not result in an indication for reoperation, with a MAD of 37.7 (33.6, 42.3), 38.6 (33, 43.8), 39.4 (31.9, 43.8), 34.4 (27.9, 38.2) and 30.7 (27, 37.6) at areas A, B, C, D and E, respectively, at the latest follow-up (Table 3). MAD at midterm CTA aortic levels B (ρ = 0.78, *P* < 0.001), C (ρ  = 0.73, *P* < 0.001), D (ρ  = 0.57 *P* = 0.007) and E (ρ = 0.57, *P* = 0.009) correlated strongly with late CTA. Complete FL collapse or thrombosis was achieved in the majority of patients, in the thoracic aorta. At the midterm time point, complete FL thrombosis in the thoracoabdominal aorta areas C, D and E was observed in 47%, 15% and 24% of available CTA and was 29%, 21% and 29% among the late follow-up CTA.

**Figure 4:**
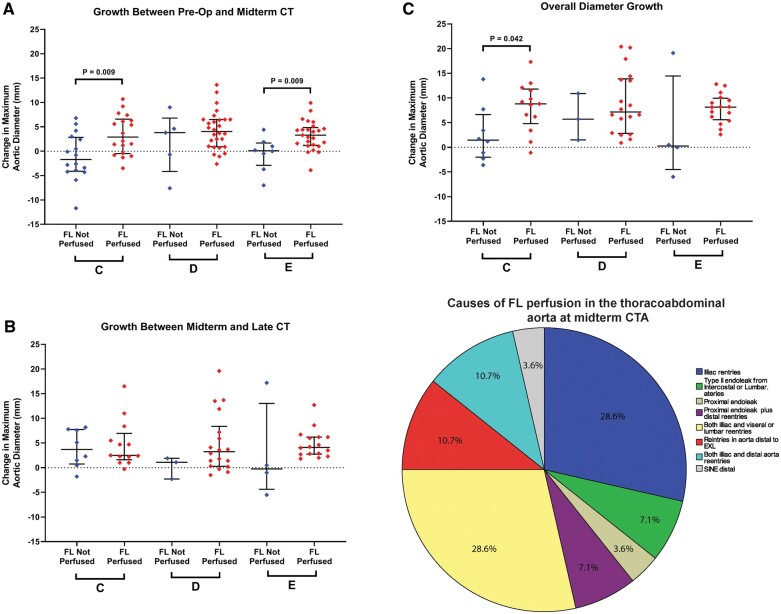
The association of persistent false lumen perfusion and aortic growth on midterm computed tomography angiography scans **(A)**, late computed tomography angiography scans **(B)** and overall **(C)**. The main causes of persistent false lumen perfusion on midterm computed tomography angiography scans **(D)**. CT: computed tomography; CTA: computed tomography angiography; FL: false lumen; Pre-op: preoperative.

Positive aortic remodelling at midterm was seen among 31 (94%), 11(32%) and 6 (18%) patients in the thoracic, thoracoabdominal and infrarenal regions, respectively. Late follow-up complete positive aortic remodelling was observed in 18 (72%) patients in the thoracic aorta, in 10 (41.7%) patients in the thoracoabdominal aorta and in 6 (25%) in the infrarenal region. Among the 28 patients with ongoing FL perfusion at midterm, 19 (68%) patients had iliac re-entries either alone or in combination with re-entries from visceral and lumber arteries or otherwise the infra-abdominal aorta (Fig. 4D).

### Statistical associations of computed tomography angiography parameters with outcomes

Higher preoperative MAD at aortic levels B (HR 1.16, 95% CI 1.02–1.31, *P* = 0.022) and E (HR 1.21, 95% CI 1.1–1.34, *P* < 0.001) was associated with an increased risk for distal aortic reintervention including thoracoabdominal replacement, abdominal aortic replacement, EVAR and distal extensions. The optimal cutoffs based on Youden’s J index for predicting distal aortic reintervention were 41.6 mm (sensitivity = 64%, specificity = 79%) at level B and 27 mm (sensitivity = 75%, specificity = 73%) at level E. Moreover a higher MAD at midterm CTA at levels D (HR 7.26, 95% CI 2.41–21.9, *P* < 0.001) and E (HR 4.53, 95% CI 1.19–17.4, *P* = 0.027) also predicted increased risk of distal aortic interventions (Fig. 6) with a Youden′s index cutoff of 33.5 mm (sensitivity = 75%, specificity = 83%) and 29 mm (sensitivity = 75%, specificity = 79%), respectively.

A lower postoperative TLI at aortic level A (HR 0.73, 95% CI: 0.59–0.91, *P* = 0.004) was protective against distal aortic reinterventions (Fig. 6). Lower TLI at aortic levels A (HR 0.71, 95% CI 0.54–0.94, *P* = 0.015) and B (HR 0.56, 95% CI 0.33–0.94, *P* = 0.029) on midterm CTA were protective against overall aortic reinterventions (Fig. 2C).

**Figure 5:**
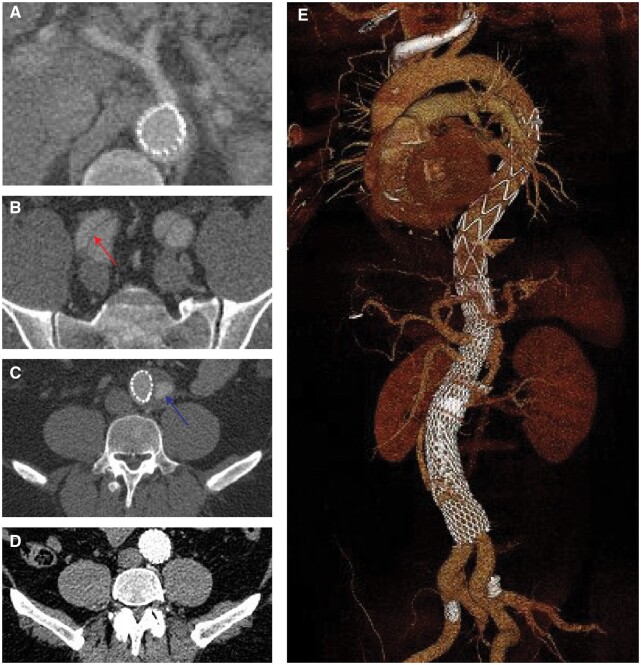
An example from 1 patient showing the progress of aortic remodelling following the Petticoat technique. **(A)** Subtotal true lumen collapse at the coeliac level preoperatively. **(B)** The true lumen is well perfused postoperatively but with **(C)** bilateral iliac re-entries leading to retrograde perfusion **(D)** in the distal aorta. **(E)** Complete positive aortic remodelling at 7 years postoperatively after early closure of the bilateral iliac re-entries.

Overall, patients with persistent FL perfusion on midterm CTA also had higher aortic growth preoperatively compared to 1-year and late-term MAD. This correlation was significant for the 2 time points at aortic area C and for the change between preoperatively and at 1 year for aortic area E (Fig. 4). Fig. 5 demonstrates the progression of aortic remodelling using a case example where closure of iliac re-entries 5 months postoperatively led to positive remodelling on follow-up 7 years later.

**Figure 6:**
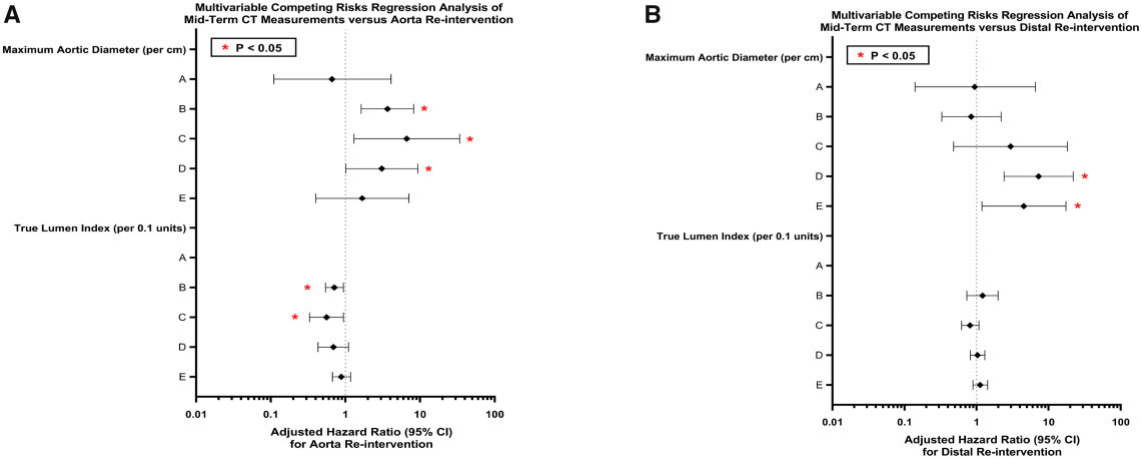
The association of maximum aortic diameter and true lumen index on overall aortic reintervention **(A)** and distal aortic reintervention **(B)**.

## DISCUSSION

Type B aortic dissection involving the entire thoracoabdominal aorta poses a risk for visceral or iliac malperfusion in the acute phase and predisposes the patient to aneurysmal dilation of the distal aorta after initial treatment with TEVAR. The PETTICOAT technique has been used as an adjutant to endovascular repair for type B dissection for more than 10 years [7–10]. The basic idea is to support the distal intima beyond the place where a covered stent graft can safely be implanted. Among patients with visceral malperfusion or those with inadequate true lumen perfusion following TEVAR, the advantages of this approach in the short term are clear. However, the indications for this technique in patients without malperfusion as a way to promote improved remodelling in the long term have remained controversial because not all patients undergoing TEVAR with the PETTICOAT technique achieve positive long-term aortic remodelling [19].

Sultan *et al.* reported improved true lumen diameters and true lumen ratios of the abdominal aorta at the 6-month follow-up, among patients with type B aortic dissection undergoing the PETTICOAT technique compared to those undergoing TEVAR alone [20]. Similarly a recent analysis by Nienaber and colleagues of the patients from the ASSIST study found significantly more false lumen thrombosis in the abdominal aorta among patients undergoing the PETTICOAT technique compared to propensity matched patients who underwent TEVAR alone [17]. These results are similar to those found by Hashizume *et al.* and Huang *et al.* in their cohorts of patients [16, 21]. However, other studies have reached the opposite conclusions. Mascia and colleagues observed aneurysm formation among 31% of patients at a medium of 85 months of follow-up [22]. There has to our knowledge been no substantive evaluation of the factors associated with distal aortic aneurysmal growth in the mid to long term following TEVAR and the PETTICOAT technique. As one of the early adaptors of the PETTICOAT concept, our centre has accumulated sufficient patient numbers to start to approach this question.

In our cohort 65 patients with acute or subacute type B dissections treated with TEVAR and the PETTICOAT technique, 45% of patients had thoracic stent graft landing zones proximal to the left subclavian artery takeoff, and 49 patients had direct visceral or iliac stents implanted. Complete false lumen thrombosis at the coeliac trunk, renal arteries and aortic bifurcation levels was observed in 47%, 15% and 24% of patients at midterm and in 29%, 21% and 29% on late CTA. Persistent FL perfusion of the distal aorta at midterm is caused mainly by iliac, visceral, lumber and distal aorta re-entries. Patients with persistent FL perfusion have an increased risk for aortic aneurysmal growth at late follow-up. A larger abdominal aortic diameter at midterm was associated with an increased probability of distal aortic reinterventions. The foregoing findings point out that ongoing FL perfusion following the PETTICOAT technique may be an important contributing factor to late aneurysmal growth.

The E-XL stent (Jotec GmbH) has been our preferred bare-metal stent over the past decade. However, the manufacturer no longer carries this device. We have adjusted our practice to using the Zenith Dissection Endovascular stent (Cook Medical, Bloomington, IN, USA) . Both stent types have been described for use with the Petticoat technique but have some differences in workflow and sizing considerations.

### Limitations

This is a single-centre retrospective study. Midterm follow-up CTA were only available for 34 patients, reducing the statistical power of this already limited number of patients.

### Conclusions

Following TEVAR with the PETTICOAT technique for acute and subacute type-B aortic dissection, on-going false lumen perfusion and higher aortic diameters, especially at the midterm CTA, have important prognostic value for aortic growth and distal aortic reintervention on long-term follow-up. In the majority of cases, persistent false lumen perfusion was caused by iliac or other distal re-entries.

## Data Availability

All relevant data are within the manuscript and its supporting information files.

## References

[1] Erbel R , Aboyans V, Boileau C, Bossone E, Di Bartolomeo R, Eggebrecht H et al 2014 ESC guidelines on the diagnosis and treatment of aortic diseases. Eur Heart J 2014 Nov 1;35(41):2873–96. https://10.1093/eurheartj/ehu281. 25173340 10.1093/eurheartj/ehu281

[2] Nienaber CA , Rousseau H, Eggebrecht H, Kische S, Fattori R, Rehders TC et al Randomized comparison of strategies for type B aortic dissection: the INvestigation of STEnt grafts in aortic dissection (INSTEAD) trial. Circulation 2009;120:2519–28. https://10.1161/CIRCULATIONAHA.109.886408. 19996018 10.1161/CIRCULATIONAHA.109.886408

[3] Fattori R , Montgomery D, Lovato L, Kische S, Di Eusanio M, Ince H et al Survival after endovascular therapy in patients with type b aortic DISSECTION: a report from the international registry of acute aortic dissection (IRAD). JACC Cardiovasc Interv 2013;6(8):876–82; https://10.1016/j.jcin.2013.05.003. 10.1016/j.jcin.2013.05.003 23968705

[4] Conrad MF , Carvalho S, Ergul E, Kwolek CJ, Lancaster RT, Patel VI et al Late aortic remodeling persists in the stented segment after endovascular repair of acute complicated type B aortic dissection. J Vasc Surg 2015;62(3):600–5. https://10.1016/j.jvs.2015.03.064. 10.1016/j.jvs.2015.03.064 26054588

[5] Pantaleo A , Jafrancesco G, Buia F, Leone A, Lovato L, Russo V et al Distal Stent Graft-Induced New Entry: an Emerging Complication of Endovascular Treatment in Aortic Dissection. Ann Thorac Surg 2016;102:527–32. https://10.1016/j.athoracsur.2016.02.001. 27112653 10.1016/j.athoracsur.2016.02.001

[6] Famularo M , Meyermann K, Lombardi JV. Aneurysmal degeneration of type B aortic dissections after thoracic endovascular aortic repair: a systematic review. J Vasc Surg 2017;66:924–30. https://10.1016/j.jvs.2017.06.067. 28736120 10.1016/j.jvs.2017.06.067

[7] Nienaber CA , Kische S, Zeller T, Rehders TC, Schneider H, Lorenzen B et al Provisional extension to induce complete attachment after stent-graft placement in type B aortic dissection: the PETTICOAT concept. J Endovasc Ther 2006;13(6):738–46. https://10.1583/06-1923.1. 10.1583/06-1923.1 17154712

[8] Lombardi JV , Cambria RP, Nienaber CA, Chiesa R, Teebken O, Lee A, STABLE investigators et al Prospective multicenter clinical trial (STABLE) on the endovascular treatment of complicated type B aortic dissection using a composite device design. J Vasc Surg 2012;55:629–640.e2. https://10.1016/j.jvs.2011.10.022. 22169668 10.1016/j.jvs.2011.10.022

[9] He H , Yao K, Nie WP, Wang Z, Liang Q, Shu C et al Modified Petticoat Technique with Pre-placement of a Distal Bare Stent Improves Early Aortic Remodeling after Complicated Acute Stanford Type B Aortic Dissection. Eur J Vasc Endovasc Surg 2015;50(4):450–9. https://10.1016/j.ejvs.2015.04.035 10.1016/j.ejvs.2015.04.035 26100449

[10] Molinari AC , Leo E, Ferraresi M, Ferrari SA, Terzi A, Sommaruga S et al Distal Extended Endovascular Aortic Repair PETTICOAT: a Modified Technique to Improve False Lumen Remodeling in Acute Type B Aortic Dissection. Ann Vasc Surg 2019;59:300–305. https://10.1016/j.avsg.2019.02.053. 31075476 10.1016/j.avsg.2019.02.053

[11] Kazimierczak A , Rynio P, Jędrzejczak T, Samad R, Rybicka A, Gutowski P. Aortic Remodeling After Extended PETTICOAT Technique in Acute Aortic Dissection Type III B. Ann Vasc Surg 2020;66:183–192. https://10.1016/j.avsg.2019.10.056. 31669476 10.1016/j.avsg.2019.10.056

[12] Hofferberth SC , Foley PT, Newcomb AE, Yap KK, Yii MY, Nixon IK et al Combined proximal endografting with distal bare-metal stenting for management of aortic dissection. Ann Thorac Surg 2012;93:95–102. https://10.1016/j.athoracsur.2011.06.106. 22133900 10.1016/j.athoracsur.2011.06.106

[13] Melissano G , Bertoglio L, Rinaldi E, Civilini E, Tshomba Y, Kahlberg A et al Volume changes in aortic true and false lumen after the “pETTICOAT” procedure for type B aortic dissection. J Vasc Surg 2012;55:641–51. https://10.1016/j.jvs.2011.10.025. 22285874 10.1016/j.jvs.2011.10.025

[14] Sobocinski J , Lombardi JV, Dias NV, Berger L, Zhou Q, Jia F et al Volume analysis of true and false lumens in acute complicated type B aortic dissections after thoracic endovascular aortic repair with stent grafts alone or with a composite device design. J Vasc Surg 2016;63:1216–24. https://10.1016/j.jvs.2015.11.037. 26806523 10.1016/j.jvs.2015.11.037

[15] Sultan I , Dufendach K, Kilic A, Bianco V, Trivedi D, Althouse AD et al Bare Metal Stent Use in Type B Aortic Dissection May Offer Positive Remodeling for the Distal Aorta. Ann Thorac Surg 2018;106(5):1364–1370. https://10.1016/j.athoracsur.2018.06.042. 10.1016/j.athoracsur.2018.06.042 30077594

[16] Huang CY , Hsu HL, Chen PL, Kuo TT, Chen IM, Hsu CP et al Aortic remodeling after hybrid provisional extension to induce complete attachment aortic repair of chronic residual type I aortic dissection. J Thorac Cardiovasc Surg 2019;158(4):1007–1016. https://10.1016/j.jtcvs.2018.12.041. 10.1016/j.jtcvs.2018.12.041 30773384

[17] Nienaber CA , Yuan X, Aboukoura M, Blanke P, Jakob R, Janosi RA et al Improved Remodeling With TEVAR and Distal Bare-Metal Stent in Acute Complicated Type B Dissection. Ann Thorac Surg 2020;110:1572–9. https://10.1016/j.athoracsur.2020.02.029. 32205112 10.1016/j.athoracsur.2020.02.029

[18] Bertoglio L , Rinaldi E, Melissano G, Chiesa R. The PETTICOAT concept for endovascular treatment of type B aortic dissection. J Cardiovasc Surg (Torino) 2019;60:91–9. https://10.23736/S0021-9509.17.09744-0. 10.23736/S0021-9509.17.09744-0 28183174

[19] Grabenwöger M , Alfonso F, Bachet J, Bonser R, Czerny M, Eggebrecht H et al Thoracic endovascular aortic repair (TEVAR) for the treatment of aortic diseases: a position statement from the european association for cardio-thoracic surgery (EACTS) and the european society of cardiology (ESC), in collaboration with the european assoc. Eur J Cardio-Thoracic Surg 2012;33(13):1558–63. https://10.1093/ejcts/ezs107. 10.1093/eurheartj/ehs074 22561257

[20] Sultan I , Dufendach K, Kilic A, Bianco V, Trivedi D, Althouse AD et al Bare Metal Stent Use in Type B Aortic Dissection May Offer Positive Remodeling for the Distal Aorta. Ann Thorac Surg 2018;106:1364–70. https://10.1016/j.athoracsur.2018.06.042. 30077594 10.1016/j.athoracsur.2018.06.042

[21] Hashizume K , Honda M, Mori M, Yagami T, Takaki H, Matsuoka T et al Full PETTICOAT in acute type B aortic dissection with patent false lumen may offer positive remodeling for the distal aorta. Gen Thorac Cardiovasc Surg 2021;69:926–33. https://10.1007/s11748-020-01548-3. 33205264 10.1007/s11748-020-01548-3

[22] Mascia D , Rinaldi E, Salvati S, Melloni A, Kahlberg A, Bertoglio L et al Thoracic Endovascular Aortic Repair With Additional Distal Bare Stents in Type B Aortic Dissection Does Not Prevent Long-Term Aneurysmal Degeneration. J Endovasc Ther 2021;28:425–33. https://10.1177/15266028211007459. 33834907 10.1177/15266028211007459

